# Unveiling Multi‐Scale Architectural Features in Single‐Cell Hi‐C Data Using scCAFE

**DOI:** 10.1002/advs.202416432

**Published:** 2025-04-24

**Authors:** Fuzhou Wang, Jiecong Lin, Hamid Alinejad‐Rokny, Wenjing Ma, Lingkuan Meng, Lei Huang, Jixiang Yu, Nanjun Chen, Yuchen Wang, Zhongyu Yao, Weidun Xie, Ka‐Chun Wong, Xiangtao Li

**Affiliations:** ^1^ Department of Computer Science City University of Hong Kong Kowloon Tong 000000 Hong Kong SAR; ^2^ Department of Computer Science The University of Hong Kong Pok Fu Lam 000000 Hong Kong SAR; ^3^ Molecular Pathology Unit Center for Cancer Research Massachusetts General Hospital Department of Pathology Harvard Medical School Boston MA 02129 USA; ^4^ BioMedical Machine Learning Lab Graduate School of Biomedical Engineering University of New South Wales Sydney 2052 Australia; ^5^ School of Artificial Intelligence Jilin University Changchun 132000 China; ^6^ Shenzhen Research Institute City University of Hong Kong Shenzhen 518057 China

**Keywords:** chromatin loops, compartments, single‐cell Hi‐C, TLDs

## Abstract

Single‐cell Hi‐C (scHi‐C) has provided unprecedented insights into the heterogeneity of 3D genome organization. However, its sparse and noisy nature poses challenges for computational analyses, such as chromatin architectural feature identification. Here, scCAFE is introduced, which is a deep learning model for the multi‐scale detection of architectural features at the single‐cell level. scCAFE provides a unified framework for annotating chromatin loops, TAD‐like domains (TLDs), and compartments across individual cells. This model outperforms previous scHi‐C loop calling methods and delivers accurate predictions of TLDs and compartments that are biologically consistent with previous studies. The resulting single‐cell annotations also offer a measure to characterize the heterogeneity of different levels of architectural features across cell types. This heterogeneity is then leveraged to identify a series of marker loop anchors, demontrating the potential of the 3D genome data to annotate cell identities without the aid of simultaneously sequenced omics data. Overall, scCAFE not only serves as a useful tool for analyzing single‐cell genomic architecture, but also paves the way for precise cell‐type annotations solely based on 3D genome features.

## Graphical abstract

scCAFE is a deep learning framework designed to identify multi‐scale 3D genome architectural features from single‐cell Hi‐C data without dense imputation. It predicts chromatin loops, TAD‐like domains, and A/B compartments, enabling efficient characterization of organization at the single‐cell level. scCAFE also identifies marker loop anchors, offering potential for precise cell identity annotation using 3D genome features.

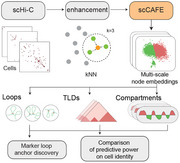

## 1 Introduction

Chromatin architecture plays a crucial role in gene regulation and cellular function. High‐throughput chromosome conformation capture (Hi‐C)^[^ ^1^, ^2^ ^]^ has significantly enhanced our understanding of 3D genome organization by providing comprehensive maps of chromatin interactions throughout the entire genome. Although the molecular basis and driving mechanisms of 3D chromatin folding are not yet fully understood, extensive research has reached a consensus that the genome is organized hierarchically in 3D space.^[^ ^3^, ^4^, ^5^ ^]^ This hierarchical organization can be broadly categorized into three layers: compartments, topologically associating domains (TADs), and chromatin loops. These layers of organizational units are ubiquitous features present throughout the whole genome^[^ ^4^, ^6^ ^]^ and have been revealed to perform critical functions in various biological processes, such as DNA replication^[^ ^7^ ^]^ and gene regulation.^[^ ^8^, ^9^ ^]^ The understanding on the multiple levels of genome architecture largely comes from the population‐level mapping of genomic loci proximity using Hi‐C‐like techniques (e.g., bulk Hi‐C). From the contact maps obtained from these assays, the architectural features exhibit distinct patterns and therefore can be easily identified using computational methods.^[^ ^6^, ^10^ ^]^

However, the patterns observed from these experiments only reflect an ensemble of the genome organization in all the individual cells. This means that the data represents an average view, masking the heterogeneity and unique configurations present across individual cells. To overcome this limitation, single‐cell Hi‐C (scHi‐C) has emerged as a powerful technique, facilitating the examination of genome architecture at the single‐cell level.^[^ ^11^, ^12^, ^13^, ^14^, ^15^, ^16^, ^17^ ^]^ scHi‐C data offers unparalleled insights into the heterogeneity of chromatin organization among individual cells, unveiling distinct structural configurations that are typically obscured in bulk analyses. This single‐cell lens is especially vital for understanding the dynamic nature of chromatin interactions in various biological contexts,^[^ ^18^ ^]^ including development,^[^ ^19^ ^]^ differentiation,^[^ ^20^ ^]^ and aging throughout the lifespan.^[^ ^17^ ^]^ However, the sparse and noisy characteristics of scHi‐C data present substantial challenges for computational analysis, that the visual patterns typically observed in bulk Hi‐C contact maps are significantly diminished in scHi‐C data. This has necessitated the development of robust methods specifically designed to detect architectural features in scHi‐C data.

Multiple previous studies have converged on a paradigm for scHi‐C architectural feature analysis, which can be summarized as an “imputation‐and‐calling” strategy. First, the contact maps are imputed to a density equivalent to bulk levels to recover the visual patterns of the architectural features. Then, algorithms that operate on the dense contact maps are applied.^[^ ^21^, ^22^, ^23^, ^24^, ^25^, ^26^, ^27^ ^]^ Despite the remarkable performance these methods offer, this strategy often demands substantial computational resources or extended execution time. Moreover, the reliance on imputation may introduce biases and limit the achievable resolution, potentially obscuring fine‐grained architectural features. To facilitate a more efficient identification of architectural features at the single‐cell level, several tools have been published recently for single‐cell loop detection^[^ ^28^ ^]^ and single‐cell TAD‐like‐domain (TLD) identification.^[^ ^29^, ^30^ ^]^ However, to the best of our knowledge, there has not been an integrative framework capable of predicting all three levels of 3D genome features at the single‐cell level without relying on imputation. This absence can increase the complexity and difficulty of practical applications. Additionally, an integrated framework for multi‐layer prediction could provide a unified understanding of the dynamics of architectural features, offering a comprehensive view of spatial genome organization. With the multi‐scale representation extracted, such an approach has the potential to reveal novel insights into the intricate relationship between 3D genome features and cellular functions.

In this study, we introduce scCAFE (Calling Architectural FeaturEs at the single‐cell level), a comprehensive framework that utilizes multi‐task learning techniques to predict 3D architectural elements from scHi‐C data without relying on dense imputation. scCAFE is designed to simultaneously predict chromatin loops and reconstruct sparse contact maps, thereby generating a highly expressive representation of genome organization. Following the acquisition of these embeddings, we apply unsupervised learning methods to identify TLDs and compartments. scCAFE surpasses existing state‐of‐the‐art methods in architectural feature calling. Leveraging the feature calls generated by scCAFE, we assess the predictive capabilities of various layers of single‐cell architectural features in determining cell identity. Our findings reveal that compartments and loops serve as better predictors of cell identity compared to TLDs. Additionally, we investigate the feasibility of identifying cell types based solely on loop frequencies of each loop anchor. These insights underscore the potential of scCAFE in advancing our understanding of genome organization and its implications for cellular differentiation and identity.

## 2 Results

### 2.1 Constructing Machine Learning Models to Detect Single‐Cell 3D Architectural Features

**Figure 1:**
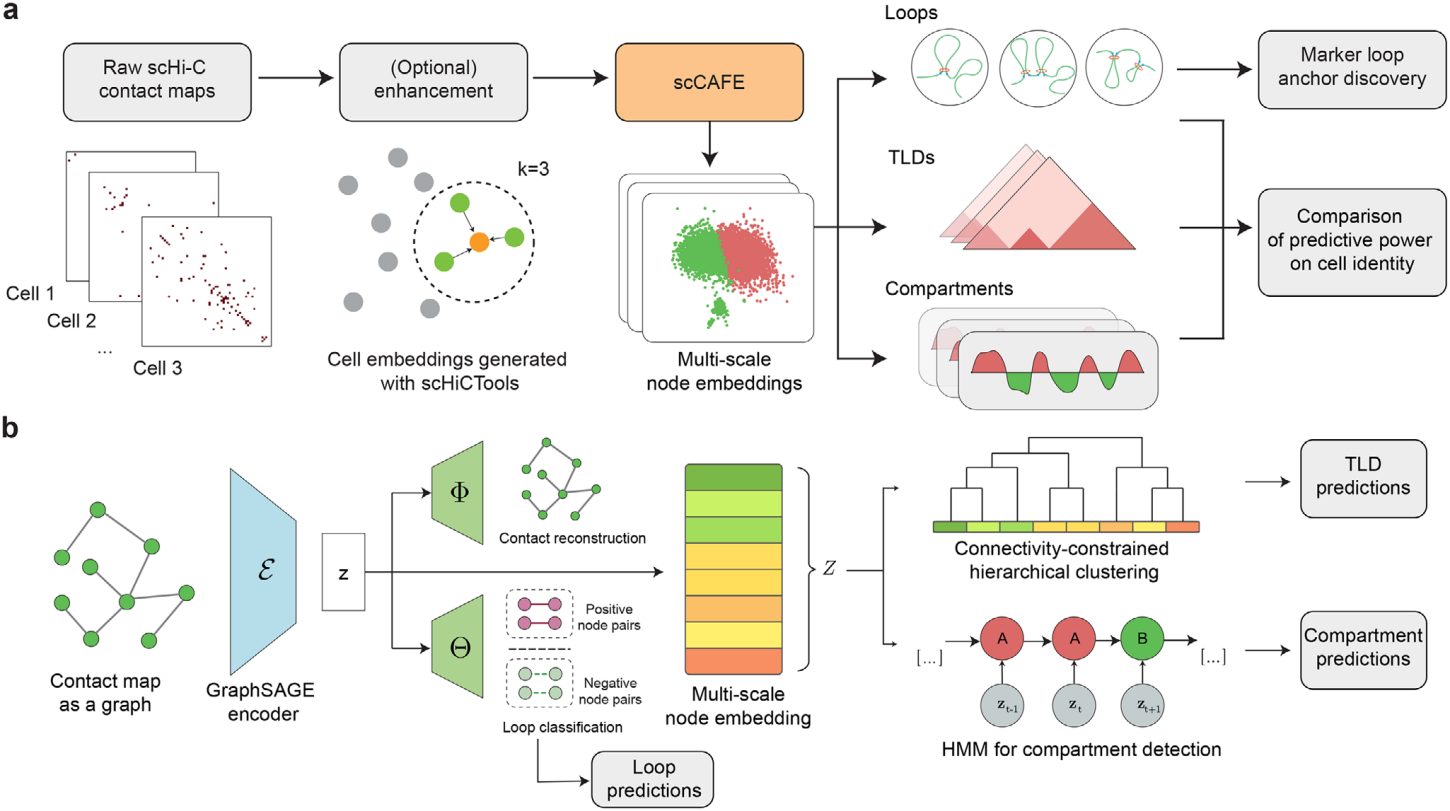
Overview of the scCAFE framework. a) Workflow of scCAFE. b) Model architecture of scCAFE. Each input contact map is treated as a graph and passed through a GraphSAGE encoder to generate latent variables. These latent features are then decoded by two decoders, Φ and Θ, to reconstruct the original contact maps and classify the loops, respectively. Subsequently, the latent features are treated as an ordered sequence. They are input to a connectivity‐constrained hierarchical clustering model for TLD predictions and fed to a hidden Markov model (HMM) for compartment predictions.

To detect 3D architectural features from the extremely sparse single‐cell Hi‐C data without densely imputing the contact maps, we designed a neural network with multi‐task learning techniques. We illustrated the overall architecture of scCAFE in **Figure** **1**. The multi‐task variational graph autoencoder (VGAE) proposed in this study was trained to simultaneously predict chromatin loops and reconstruct contact maps. This approach enabled the model to learn latent features that effectively captured both loop patterns and the global 3D organizational features of chromosomes. The representations learned via this method were beneficial for predicting chromatin loops and enabled the unsupervised identification of TLDs and compartments at the single‐cell level.

In the framework of scCAFE, the chromosome graphs were not densified to recover the data resolution of bulk Hi‐C data. Instead, only graphs of poor quality were slightly enhanced using an enhancement module. Despite this enhancement, the contact maps remained sparse, maintaining the computational efficiency of the model. The model's predictive efficacy for multi‐scale organizational units (introduced in later sections) also validated that 3D architectural features could be directly inferred from the sparse contact matrices without the need for dense imputation.

An important principle we followed in constructing the model was the multi‐view nature of Hi‐C data.^[^ ^28^, ^31^ ^]^ Consistent with scGSLoop,^[^ ^28^ ^]^ in scCAFE, the neural network operates on the graph view generated from contact maps, with node (genomic bin) features derived from DNA sequences, reflecting the sequence view.^[^ ^32^, ^33^ ^]^ Moreover, we leveraged the sequence characteristics of the data to predict higher‐order organizational units at the single‐cell level, including TLDs and compartments. Given that the genomic bins have an order on the linear genome, the node embeddings of a chromosome generated by the neural network can be considered as a linearly connected sequence, which allowed us to apply sequence‐based learning algorithms effectively. In particular, hierarchical clustering with a linear connectivity constraint was adopted for TLD calling, while an HMM was utilized for compartment identification. The detailed approach is outlined later in the Methods section.

A simple yet effective method for marker loop anchor identification was proposed and integrated into scCAFE. By utilizing chromatin loops detected at the single‐cell level, scCAFE recognizes 10 kb‐genomic bins that are significantly enriched with chromatin loops in different cell types. Except for single‐cell predictions of 3D genome features at different scales, the scCAFE model also provides an interface to predict these features at the consensus level. This dual capability enables researchers to investigate the spatial structures of the genome both within individual cells and across cell populations, offering a comprehensive view of 3D genomic organization.

### 2.2 scCAFE Predicts Loops Accurately for Both Single Cells and Cell Populations

**Figure 2:**
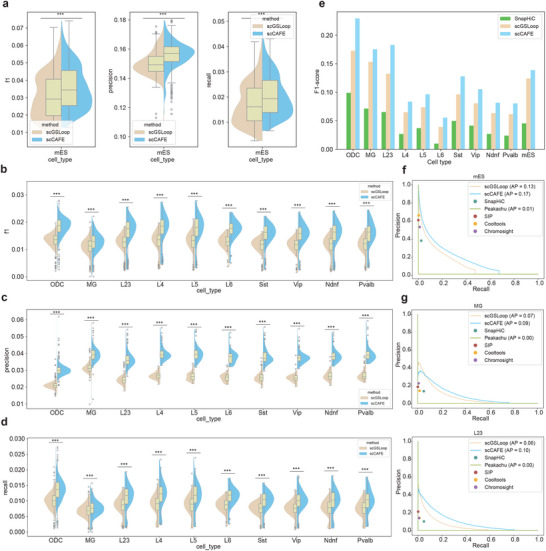
scCAFE accurately detects chromatin loops at both the single‐cell level and the consensus level. a) F1 scores, precisions, and recalls of single‐cell chromatin loops in the mESC dataset predicted by scCAFE and scGSLoop. Three asterisks (***) indicate a p‐value smaller than 0.001, determined using the Wilcoxon signed‐rank test (the same in panels b–d). b–d) F1 scores, precisions, and recalls of single‐cell loops in different cell types of the hPFC dataset, compared between scCAFE and scGSLoop. e) F1 scores for consensus loops across different cell types in both datasets, comparing the performance of SnapHiC, scGSLoop, and scCAFE. f) Precision‐recall plots of the consensus loops in the mESC dataset predicted by scGSLoop, scCAFE, SnapHiC, Peakachu, SIP, Cooltools, and Chromosight, respectively. g) Precision‐recall plots of the consensus loops in MG and L2/3 cells predicted by scGSLoop, scCAFE, SnapHiC, Peakachu, SIP, Cooltools, and Chromosight, respectively. The precision‐recall curves in other cell types of the hPFC dataset can be found in Figure S2 (Supporting Information).

We conducted a comprehensive assessment of scCAFE's performance in predicting chromatin loops at both the single‐cell and consensus levels. The datasets that we used for performance evaluation include the mouse embryonic cells (mESC) dataset from ref.[12] and the human prefrontal cortex (hPFC) dataset from ref. [34]. First, we compared our method against scGSLoop^[^ ^28^ ^]^ to evaluate its ability in identifying loops at the single‐cell level. The distributions of single‐cell loop sizes and loop counts for scCAFE across different datasets are provided in Figure S1 (Supporting Information). For each cell type, the F1 score, precision, and recall were calculated for each individual cell using the single‐cell loop calls. The results of the comparisons in terms of these metrics are presented in **Figure** **2**a–d, illustrating the effectiveness of the model in accurately predicting single‐cell loops. By conducting Wilcoxon signed‐rank tests on the F1 scores, precisions, and recalls of scGSLoop and scCAFE, it was shown that scCAFE significantly outperformed scGSLoop in all the cell types of the two datasets (denoted by the asterisks in Figure 2a–d).

In certain studies, researchers focus particularly on the chromatin loops of various cell types and sub‐types. Consequently, it is crucial that a single‐cell loop‐calling method is capable of predicting chromatin loops summarizing the average looping patterns of the cell population. In scCAFE, we adopted the same aggregation method as in scGSLoop to predict consensus loops (i.e., the loops at the population level). It was demonstrated that the consensus loop predictions consistently achieved higher F1 scores compared to SnapHiC and scGSLoop across all tested cell types (Figure 2e). Additionally, in order to comprehensively examine the performance of scCAFE in terms of consensus loop prediction, we conducted additional evaluations across various probability thresholds using PR curves. For this comparison, we included multiple mainstream loop calling tools developed for either bulk or single‐cell Hi‐C data, including scGSLoop,^[^ ^28^ ^]^ SnapHiC,^[^ ^22^, ^25^ ^]^ SIP,^[^ ^35^ ^]^ cooltools,^[^ ^36^ ^]^ Peakachu,^[^ ^37^ ^]^ and Chromosight.^[^ ^38^ ^]^ As shown in Figure 2f,g and Figure S2 (Supporting Information), the PR curves of scCAFE exhibited higher precision across various recall values compared to the scGSLoop model in 9 out of 11 cell types from both the mESC and hPFC datasets. In both datasets, scCAFE achieved higher average precision (AP) than scGSLoop across all cell types. The PR curves of scCAFE were also consistently higher than the PR plots of SnapHiC, SIP, Cooltools, Peakachu, and Chromosight in all the cell types. These results suggest that the algorithmic design of our method improves loop predictions at the consensus level, providing better performance than previous approaches.

In all the performance assessments described above, we adhered to the principle of training the model on one dataset and testing it on a different dataset. This approach ensures a fair comparison and better simulates real‐world applications. The remarkable performance, despite differences in data conditions and species, highlights the model's ability to generalize across varying scenarios.

### 2.3 scCAFE Facilitates Accurate Loop Detection on scNanoHi‐C Data

Third‐generation sequencing technologies, characterized by their ability to produce long reads, are poised to play a critical role in advancing genomics research. These technologies enable more accurate and comprehensive analyses of complex genomic structures, particularly in challenging contexts such as repetitive regions or degraded DNA samples. As one of the first attempts to apply third‐generation sequencing to single‐cell 3D genomics, scNanoHi‐C^[^ ^16^ ^]^ represents a significant breakthrough, providing high‐resolution chromatin conformation data at the single‐cell level. The development of computational methods capable of handling this emerging data type is essential for unlocking its full potential in understanding genome organization.

**Figure 3:**
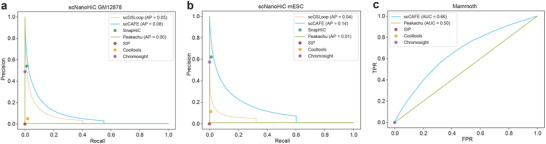
Loop calling performance of scCAFE on the scNanoHiC datasets and the ancient woolly mammoth dataset. a) PR plots comparing scCAFE and benchmark methods on scNanoHiC GM12878 data. b) PR plots comparing scCAFE and benchmark methods on scNanoHiC mESC data. c) ROC curves of scCAFE and benchmark tools on the ancient woolly mammoth dataset.

To evaluate the generalizability and robustness of scCAFE, we applied the model to two datasets generated using scNanoHi‐C technology: GM12878 and mESC.^[^ ^16^ ^]^ These datasets provide high‐resolution single‐cell 3D genome information, allowing us to assess scCAFE's ability to identify chromatin loops in data generated using an alternative sequencing technology. We compared scCAFE with state‐of‐the‐art tools, including scGSLoop,^[^ ^28^ ^]^ SnapHiC,^[^ ^22^, ^25^ ^]^ SIP,^[^ ^35^ ^]^ Peakachu^[^ ^37^ ^]^, Cooltools,^[^ ^36^ ^]^ and Chromosight.^[^ ^38^ ^]^ The evaluation was conducted using PR curves across a range of probability thresholds. As shown in **Figure** **3**a,b, scCAFE consistently outperformed all comparison tools across these thresholds in both datasets. These results highlight scCAFE's robustness and its ability to generalize to datasets derived from diverse sequencing platforms and cell types.

### 2.4 scCAFE can Predict Loops on Downgraded Hi‐C Data From Ancient DNA

An important characteristic of scHi‐C data is its extreme sparsity and noise. Similar challenges are also encountered in ancient 3D genome data.^[^ ^39^ ^]^ Unlike scHi‐C datasets, which typically consist of contact maps from multiple single cells, ancient Hi‐C data provide only a single contact map per chromosome, with sequencing depth comparable to that of a single cell in scHi‐C. Inspired by previous studies demonstrating that compartmentalization scores can be inferred from downsampled bulk Hi‐C data,^[^ ^40^ ^]^ we sought to investigate whether chromatin loops could be detected in the even more sparse and noisy ancient 3D genome data. To this end, we further extended the application of scCAFE to predict chromatin loops in mammoth ancient DNA Hi‐C data.^[^ ^39^ ^]^ Remarkably, scCAFE was the only tool among the five tested methods (SIP,^[^ ^35^ ^]^ cooltools,^[^ ^36^ ^]^ Peakachu,^[^ ^37^ ^]^ Chromosight,^[^ ^38^ ^]^ and scCAFE) that could reliably classify loop interactions in ancient DNA Hi‐C data. As shown in Figure 3c, the ROC curve highlights scCAFE's superior performance compared to other tools in this context.

Ancient DNA Hi‐C data is notoriously challenging due to the degradation of samples and the resulting high noise‐to‐signal ratio, which complicates loop detection. Despite these challenges, scCAFE demonstrated robust performance, showcasing its adaptability and effectiveness for low‐quality datasets. These findings indicate that scCAFE is not only suitable for single‐cell Hi‐C analysis but also has the potential to analyze the three‐dimensional genome organization of ancient DNA, providing a promising tool for understanding the chromatin structure of extinct species.

### 2.5 Detection of TLDs in scHi‐C Data Using scCAFE

**Figure 4:**
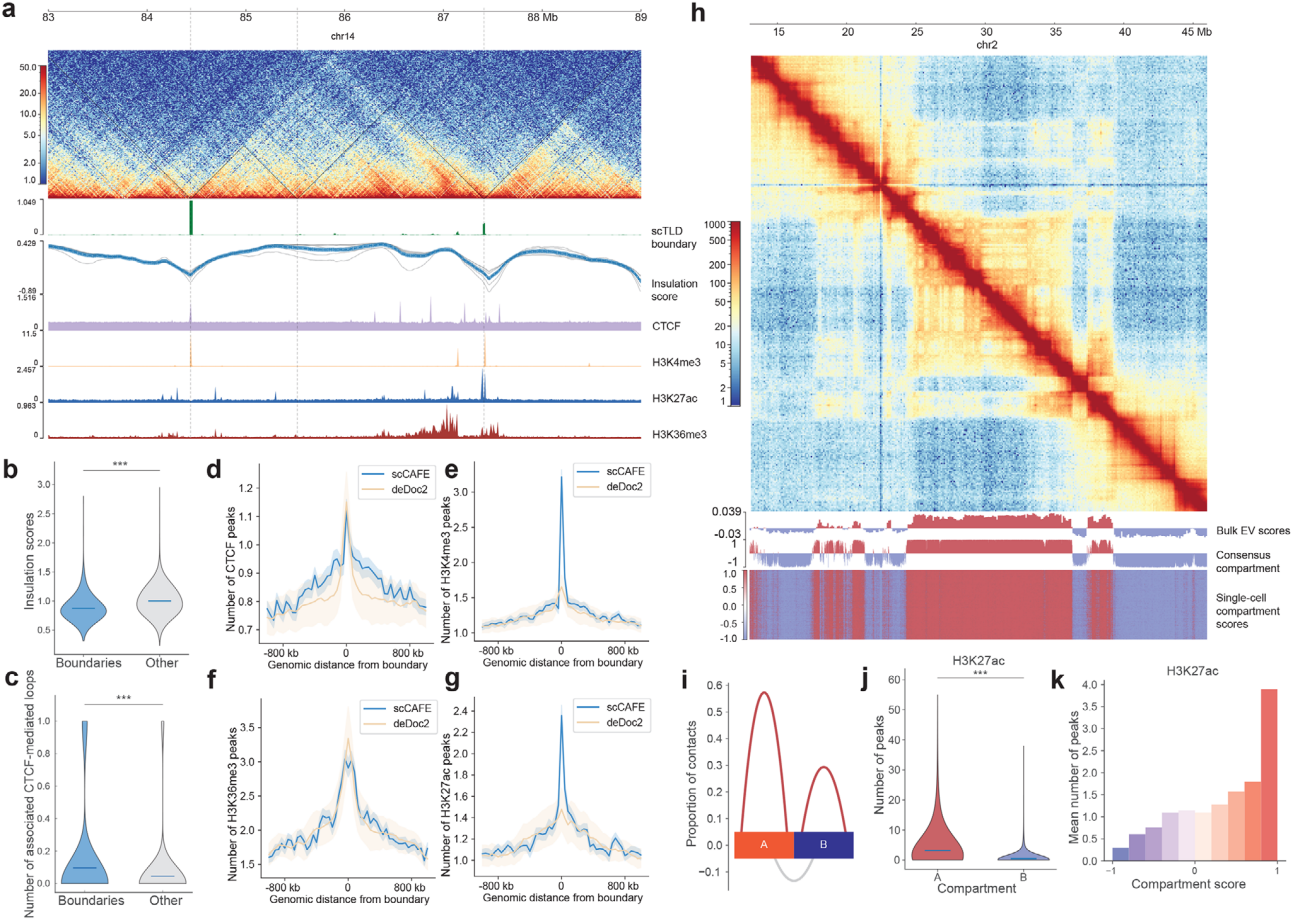
TLDs and compartments predicted by scCAFE are validated by bulk Hi‐C and epigenetic features. a) An example region in the genome of mESC, annotated with scCAFE‐predicted consensus TLDs and single‐cell TLDs. The insulation scores detected in bulk Hi‐C data, as well as the tracks of CTCF, H3K4me3, H3K27ac, and H3K36me3 ChIP‐Seq are also annotated. The predicted TLDs show consistency with the bulk insulation scores and epigenetic features. b) The bulk insulation scores at the boundary regions and non‐boundary regions. Three asterisks denote significance level of p‐value smaller than 0.001, Mann–Whitney U test (same for panel c). c) The numbers of CTCF‐mediated loops detected at boundary regions and non‐boundary regions by ChIA‐PET. (d‐g) Enrichment profiles of CTCF, H3K4me3, H3K36me3, and H3K27ac ChIP‐Seq peaks around single‐cell TLD boundaries detected by scCAFE and deDoc2. Shaded areas represent +/‐ standard deviation. h) An example genomic region showing scCAFE‐predicted single‐cell and consensus compartment scores. i) Bulk Hi‐C interactions within and between compartments. j) Numbers of H3K27ac ChIP‐Seq peaks in compartment A and B. k) Numbers of H3K27ac peaks at different scCAFE compartment scores.

scCAFE has empowered us to unveil multiple layers of 3D organizational elements within the genome through the utilization of unsupervised learning algorithms. In addition to identifying chromatin loops, we have successfully detected higher‐order elements, such as TLDs and compartments, at both the single‐cell and consensus levels. This section focuses on evaluating the performance of scCAFE in TLD calling. Specifically, we identified single‐cell TLDs and consensus TLDs in the mESC dataset. **Figure** **4**a illustrates an example region of the mESC genome, highlighting the TLDs identified by scCAFE. It is shown that the predictions generated by scCAFE at both the single‐cell level and the consensus level were highly consistent with the patterns observed in bulk Hi‐C. In particular, the single‐cell TLD and consensus TLD boundaries aligned with the local minima of insulation scores and previously reported boundary‐enriched factors, such as CTCF, H3K4me3, and H3K27ac.^[^ ^41^, ^42^ ^]^

We conducted a series of genome‐wide analyses to investigate the structural and functional characteristics of the identified TLDs. First, we compared the insulation scores between the boundary regions and the non‐boundary regions. Previous studies have shown a strong correlation between TAD boundary occurrences and lower insulation scores.^[^ ^43^ ^]^ In our analysis, the lower insulation scores derived from bulk Hi‐C data serve as a validation marker for the consensus TLD boundaries. As illustrated in Figure 4b, the insulation scores are significantly lower at the consensus boundary regions detected by scCAFE, indicating a clear demarcation of TLD boundaries.

We next investigated the enrichment of CTCF and histone modifications at these boundaries as detected by scCAFE. Figure 4d‐g demonstrate a clear enrichment of CTCF binding, H3K4me3, H3K36me3, and H3K27ac at the single‐cell TLD boundaries detected by our method. This finding aligns with the established enrichment profiles reported in previous research.^[^ ^41^ ^]^ We also compared these enrichment profiles with the ones detected using the state‐of‐the‐art single‐cell TLD calling method deDoc2.^[^ ^30^ ^]^ It is shown in Figure 4d–g that the enrichment of CTCF and H3K36me3 of scCAFE‐predicted TLD boundaries is slightly less pronounced than deDoc2 predictions. However, our model produced much more enriched profiles of H3K4me3 and H3K27ac, indicating a distinct, functionality‐related TLD pattern predicted by scCAFE. Another layer of relevant structural properties of TLD boundaries is that they usually co‐exist with CTCF‐mediated loops.^[^ ^2^, ^3^, ^44^ ^]^ Hence, we utilized an independent ChIA‐PET dataset on CTCF^[^ ^45^ ^]^ to validate our model's predicted TLD boundaries. By integrating this dataset, we were able to confirm that our consensus TLD boundaries are associated with significantly higher numbers of CTCF‐mediated loops compared with non‐boundary regions (Figure 4c).

To assess the robustness of our method in handling scHi‐C data with poorly sequenced regions, we conducted additional analyses by evaluating its performance in scenarios with artificially introduced data sparsity. Specifically, we removed all contacts within a randomly selected 1Mb genomic region for each cell of the mESC dataset to form a ΔRegions dataset and examined the impact of the deleted interactions on TLD boundary detection. The aggregated epigenetic profiles of TLD boundaries identified in the removed regions closely resembled those from the complete dataset, as shown in Figures S3b–e (Supporting Information). Moreover, we observed a high degree of overlap between the TLD boundaries detected in the datasets with and without these deletions (Figure S3a, Supporting Information). These results demonstrate that our enhancement strategy effectively compensates for missing data, maintaining boundary detection accuracy despite the sparsity of individual cells' contact maps.

Taken together, the TLDs detected by scCAFE exhibit a comprehensive range of typical organizational and functional characteristics as recognized in previous studies. The enrichment comparison between our model and the state‐of‐the‐art single‐cell TLD calling method has also highlighted its remarkable performance. Our random deletion experiments also further demonstrate the robustness of our method in handling sparse and poorly sequenced regions. These findings underscore the ability of scCAFE in identifying TLDs in scHi‐C data.

### 2.6 Identification of A/B Compartments in Single Cells Using scCAFE

We further applied an HMM on the embeddings generated by scCAFE to identify A/B compartments in single cells, which represent an architectural feature at the higher level of the 3D genome hierarchy. Using this method, we identified the single‐cell compartments and consensus compartments in the mESC dataset. Figure 4h presents an example region in the mESC genome where the checkerboard pattern can be clearly observed. By plotting the eigenvectors of the correlation matrix of bulk Hi‐C data alongside our predicted single‐cell and consensus compartments, we observed that the compartment patterns identified by scCAFE strongly coincide with the bulk eigenvectors and the patterns on the contact map.

By investigating the organizational and functional properties of the A/B compartments detected with our model, we aimed to further validate the compartment annotations produced by scCAFE. First, we elucidated the volume of chromatin contacts (from bulk Hi‐C data) within compartments A and B, as well as between different compartments. As shown in Figure 4i, the intra‐compartment interactions (the red curves) account for a higher proportion of the total contacts than the inter‐compartment interactions (the grey curve). This is consistent with the typical patterns of intra‐ and inter‐compartment interactions that have been widely validated in prior research.^[^ ^2^, ^46^ ^]^

From a functional perspective, compartment A is typically characterized by a greater abundance of active transcriptional activities, resulting in a higher number of active promoters and enhancers. Figure 4j and Figure S4a (Supporting Information) provide a comparison of the active histone modification markers (H3K4me3 and H3K27ac as detected in bulk ChIP‐Seq) in compartments A and B as detected by scCAFE. The analysis revealed a significantly higher enrichment of both markers in the compartment A loci. The enrichment of these markers were further elucidated in a stratified manner, where the mean number of ChIP‐Seq peaks was plotted against the posterior probability of the compartment predictions (Figure 4k; Figure S4b, Supporting Information). The results indicate that our predicted compartment scores (posterior probabilities) can serve as indicators of the activity levels at the respective loci. This offers a potential reference for exploring the regulatory characteristics of genomic regions in single cells.

### 2.7 Computational Efficiency of scCAFE

**Figure 5:**
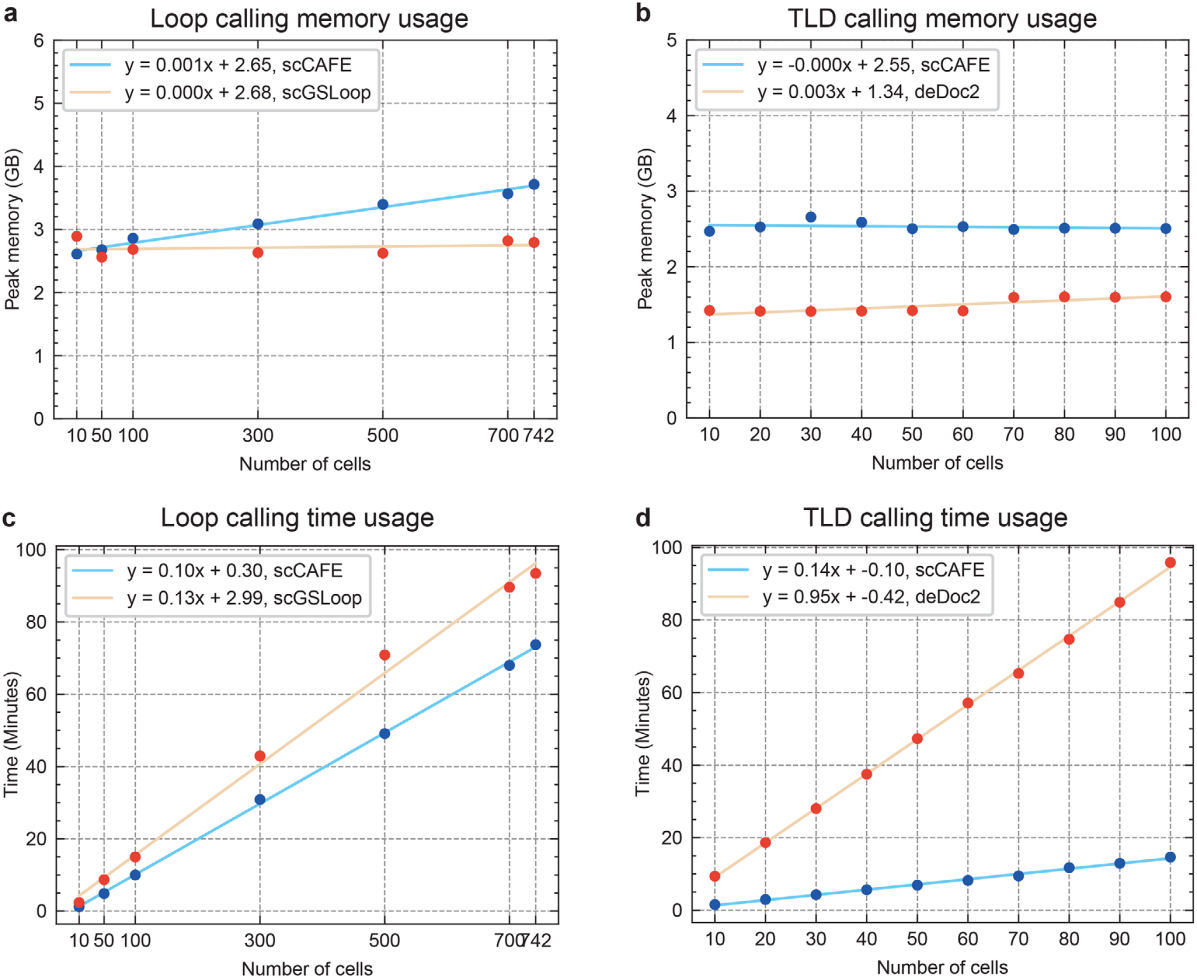
Memory and time usage of scCAFE and benchmark tools on loop calling and TLD calling. a) Loop calling memory usage of scCAFE and scGSLoop on different dataset sizes. b) TLD calling memory usage of scCAFE and deDoc2 on different dataset sizes. c) Loop calling time consumption of scCAFE and scGSLoop on different dataset sizes. d) TLD calling time consumption of scCAFE and deDoc2 on different dataset sizes.

We evaluated the computational efficiency of scCAFE by comparing its running time and memory usage with two state‐of‐the‐art tools: scGSLoop for loop calling and deDoc2 for TLD calling (**Figure** **5**). The analyses were conducted on datasets of varying sizes to assess how these metrics scale with increasing number of cells.

The peak memory usage of all tools remained relatively stable across different dataset sizes, with minimal variation. For loop calling, scCAFE exhibited a slight increase in memory usage with a slope of 0.001, while scGSLoop showed an essentially constant memory footprint (slope = 0.000). Similarly, for TLD calling, scCAFE's memory usage remained stable (slope = –0.000) and was comparable to deDoc2 (slope = 0.003). Although scCAFE's memory consumption was slightly higher than the other tools, its maximum memory usage was below 4 GB, which is well within the capacity of modern computing systems.

The running time of all tools increased linearly with the dataset size. However, scCAFE demonstrated significantly better computational efficiency, with smaller slopes compared to scGSLoop and deDoc2. For loop calling, scCAFE's slope (0.10) was lower than that of scGSLoop (0.13). For TLD calling, scCAFE's slope (0.14) was substantially smaller than that of deDoc2 (0.95). These results highlight scCAFE's ability to process data more efficiently, particularly for larger datasets.

Our analysis shows that scCAFE achieves competitive memory usage and outstanding computational efficiency compared to existing tools. The results confirm that scCAFE is well‐suited for large‐scale single‐cell data analysis, balancing memory consumption and runtime performance effectively.

### 2.8 scCAFE‐Annotated Architectural Features are Predictors of Cell Identity

scHi‐C is a powerful technique that enables the detection of cell‐to‐cell variability in the 3D genome across individual cells, as well as the heterogeneity across different cell types.^[^ ^18^, ^47^ ^]^ Several existing studies have focused on projecting scHi‐C data into a lower‐dimensional space for the purpose of cell clustering^[^ ^21^, ^23^, ^26^, ^48^ ^]^ or crafting features for cell type classification.^[^ ^47^, ^49^, ^50^ ^]^ However, the investigation of architectural features' heterogeneity across different cell types has been relatively limited. In this study, we made use of the architectural features identified by scCAFE to explore how these features are related to the cell type identity. To this end, we generated four different sets of cell features for the hPFC dataset using scCAFE: the high‐level latent features (i.e., the latent features generated by the multi‐task VGAE), loop features, TLD features, and compartment features. Although scCAFE identifies all levels of architectural elements at 10 kb resolution, we pooled the predictions to 100 kb resolution so that the feature vectors of cells could fit into memory. Using our encoding scheme, all four sets of cell features were of the same dimensionality (Methods).

**Figure 6:**
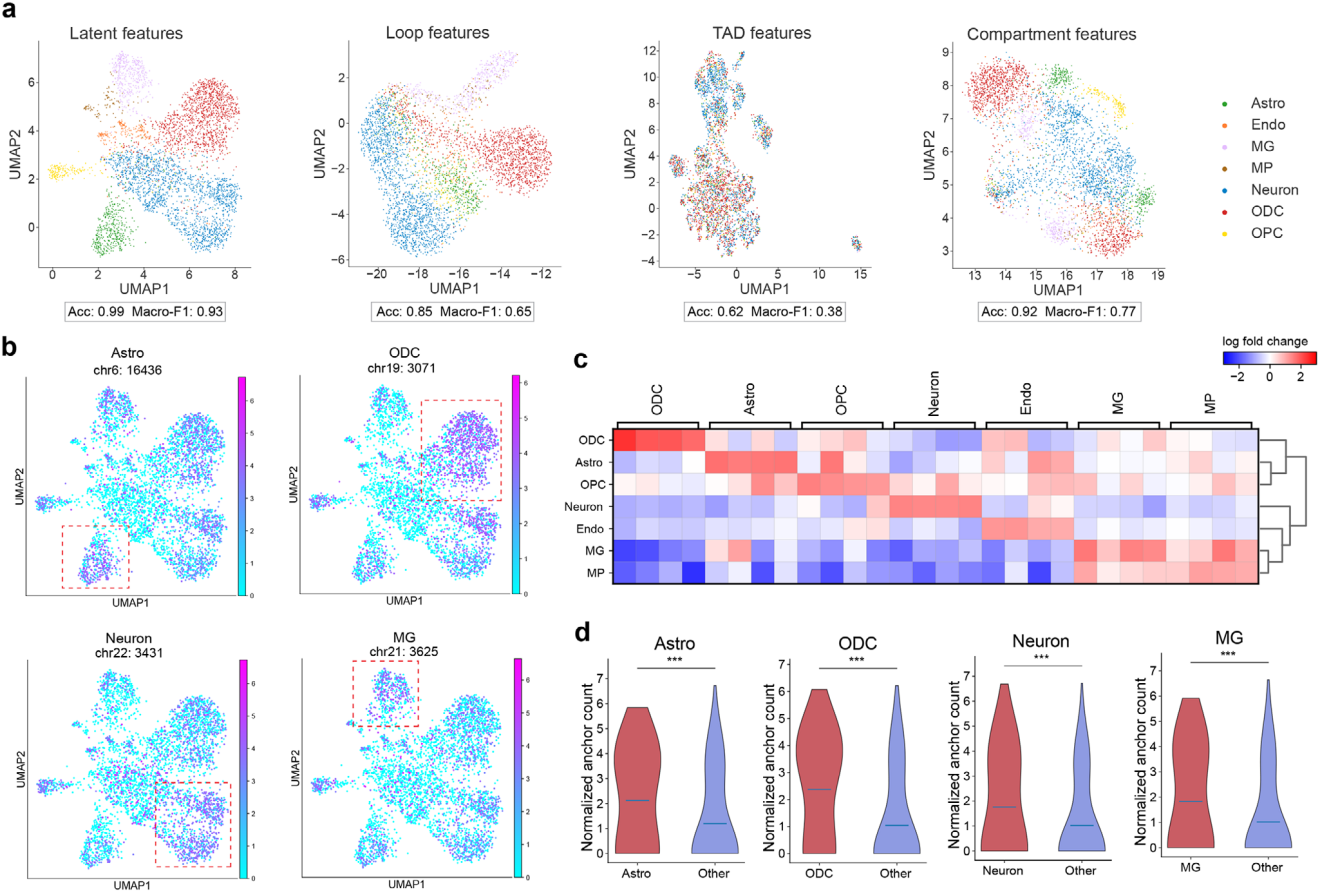
Predictive power of scCAFE‐predicted single‐cell architectural features in classifying cell types. a) UMAP plots of scCAFE latent features, single‐cell loops, TLDs, and compartments on the hPFC dataset. b) “Marker loop anchors” identified by scCAFE in Astro, ODC, Neuron, and MG. The red square in each subplot denotes the enriched target cells. c) Matrix plot of marker loop anchors. Each row represents a cell type, and each column corresponds to a loop anchor region in the genome. The color of each entry denotes the log fold change in the number of loops compared to other cell types. d) Violin plots of the loops associated with the marker loop anchors in different cell types, comparing between the enriched cell type and other cell types. Three asterisks (***) denote p‐value smaller than 0.001, Mann‐Whitney U test.

Here, we utilized unsupervised dimensionality reduction techniques to effectively visualize the cell embeddings. Additionally, we trained supervised machine learning models to assess the predictive capability of single‐cell architectural features in determining cell types. **Figure** **6**a displays the UMAP visualizations of the four sets of features and the classification performance metrics obtained using these features. The 2D visualization (Figure 6a leftmost) of the latent features clearly demonstrates the separation of the seven different cell types in the hPFC dataset. The accuracy and macro‐F1 score achieved were 0.99 and 0.93, respectively. The confusion matrix was visualized and displayed in Figure S5 (Supporting Information). These results strongly indicate that the features extracted by scCAFE successfully captured the heterogeneity of the 3D genome organizations across cell types. In comparison, the single‐cell loops and compartment features showed less capability in distinguishing between the cell types (Figure 6a; Figure S5b,d, Supporting Information). Among the four sets of features, the TLD features exhibited the least predictive power in differentiating cell types. Despite this, the TLD features still demonstrated certain classification ability, as their metrics were higher than those of a random (dummy) classifier (accuracy = 0.40, macro‐F1 = 0.08).

The observations present significant insights into the variability of genome spatial organization. Notably, the high‐level latent features exhibited superior predictive power compared to all other feature sets, as anticipated. This finding aligns with the well‐established principle of hierarchical structures within the 3D genome. In this context, different levels of single‐cell architectural features can only reflect specific aspects of the 3D genome hierarchy. On the other hand, the cell‐type heterogeneity of single‐cell TLDs was not rich enough to support accurate cell type identification, indicating a more conserved role of domain boundaries across cell types. This is in line with multiple previous studies on the conserved nature of domain boundaries, which often serve as stable organizational units within the genome.^[^ ^41^, ^51^, ^52^ ^]^ Although it has been demonstrated in Figure 4a and d–g that the TLD boundaries were variable across single cells, this heterogeneity may only account for the dynamic shifting of domain boundaries, yet not significant enough for cell type identification.

To validate the generalizability of the architectural features in discriminating cell identities, we further applied it to a scNanoHiC dataset containing three cell lines: GM12878, COLO320DM, and K562.^[^ ^16^ ^]^ The results demonstrate that the cell lines were well separated in visualized embeddings of latent, loop and compartment features (Figure S6a, Supporting Information). In supervised learning classification tasks, the latent features and compartment features consistently outperform loop and TLD features, with the latter being the least effective (Figure S6a, Supporting Information). These findings align with the conclusions from the hPFC dataset.

Overall, the degree of variability across cells differs depending on the architectural features. In the next section, we examine single‐cell loops as an example to further explore the connections between architectural features and cell identities.

### 2.9 Marker Anchors Identification Using Single‐Cell Loops Predicted by scCAFE

The concept of the marker gene has been vitally important in single‐cell analysis, as it allows for the identification and characterization of specific cell types.^[^ ^53^, ^54^, ^55^, ^56^ ^]^ In this study, we generalized the concept of marker gene to provide a visualized and interpretable profile of how the loop anchors annotate cell types.

To achieve this, we adjusted the encoding scheme for single‐cell loop anchors. The feature encoding scheme used in the former section was designed for a fair comparison between features, i.e., the feature dimensionality was identical across feature sets. This scheme encoded the features using 100 kb‐resolution bins, ensuring the resulting vectors fit into memory. This was necessary despite the fact that the original features were called at a resolution of 10 kb. To gain a better understanding of the relationship between single‐cell loops and cell types, we employed a more appropriate method to encode the single‐cell loop anchors at a 10 kb resolution, where the loop anchors in each cell were input as a text document, and the tf‐idf scheme was applied. In this way, the occurrence frequencies of loop anchors were well preserved in the resulting features.

By training seven support vector machine (SVM) models (one for each cell type) using tf‐idf features and cell type labels as inputs, we successfully developed a one‐vs‐rest multi‐class classifier capable of accurately predicting cell identity. This classifier achieved an accuracy of 0.91 and a macro‐F1 score of 0.78, indicating superior performance compared to the model using features from the previous section. By leveraging the weights learned by SVMs, we identified 50 candidate anchors for each cell type that exhibited the highest positive discriminative power. These candidate anchors were then subjected to further analysis using Scanpy^[^ ^54^ ^]^ to recognize key anchors associated with Astro, MG, Neuron, and ODC cell types. Through our analysis, we discovered key anchors that were particularly informative for distinguishing between these cell types. These key anchors, as illustrated in Figure 6b–d, exhibited a substantial variation in the number of associated loops across different cell types. This finding suggests that the distribution and abundance of loop anchors can serve as valuable markers for characterizing and differentiating distinct cell populations.

We also performed our marker loop anchor analysis on the scNanoHiC dataset, which revealed a significant enrichment of the identified loop anchors in their corresponding cell lines (Figure S6b,c, Supporting Information). This result underscores the generalizability and robustness of our method.

In summary, our study highlights the potential of loop anchors as markers for cell type identification. This may illuminate future studies in single‐cell 3D genomics by providing identification references for cell types solely from scHi‐C data.

## 3 Discussion

In this study, we proposed a computational framework scCAFE that can directly predict the architectural features of 3D genome at multiple levels without dense imputation of scHi‐C contact maps. With this framework, we were enabled to detect chromatin loops with a unprecedentedly high accuracy, and simultaneously detect the TLDs and A/B compartments that are highly consistent with the biological patterns observed in previous research. As enabled by the single‐cell architectural features detected, we characterized the heterogeneity of these features across cell types, and we discerned the predictive power of different levels of architectural elements in classifying cell identity. Another significant contribution of our study is the identification of a series of “marker loop anchors.” Although we only employed standard data science protocols for this purpose, these anchors have the potential to serve as valuable clues for cell type identification in downstream studies. This is a particularly interesting direction that might be worth exploring in the future, as previous studies have been depending on the simultaneous profiling of other omics data.^[^ ^34^, ^57^, ^58^ ^]^ This concept aligns with the differential interactions (DIs) proposed in ref. [57], which refer to the contacts that are significantly more intense in one cell type compared to others. The key distinction between these two methodologies lies in the spatial dimensionality of interest: marker loop anchors generated in our study are 1D vectors, whereas DIs produce 2D paired loci as output. Consequently, the representation of the less sparse 1D marker loop anchors is likely to exhibit greater robustness and invariance against noise.

The core principle underlying scCAFE's algorithm design also follows the multi‐view nature of Hi‐C data.^[^ ^28^, ^31^ ^]^ The embeddings for genomic bins in each single cell are extracted from the graph representation of contact maps, and the chromatin loops are also identified using the graph view. In contrast, the detection of higher‐order single‐cell architectural elements is based on the sequence view of the data, utilizing unsupervised learning techniques. Specifically, scCAFE employs multi‐task learning to capture patterns in 3D genome architecture, thereby enhancing the representational capacity of the embeddings. Hence, scCAFE can directly predict the 3D architectural features without imputing the contact maps to a density equivalent to bulk data. This strategy differs from the previously common practice in scHi‐C analysis, where sparse contact matrices are densified to identify architectural elements. By eliminating the requirement for dense imputation, this strategy provides a more efficient framework for architectural feature identification. Several recent studies have adopted this non‐imputation approach to predict single‐cell loops^[^ ^28^ ^]^ and scTLDs.^[^ ^29^, ^30^ ^]^ Nonetheless, to our knowledge, scCAFE is the first comprehensive model capable of predicting all three levels of architectural features of the 3D genome without the need for imputation.

A potential future improvement of scCAFE could be the integration of epigenomics and transcriptomics data.^[^ ^59^ ^]^ This would allow for the illumination of the connections between epigenetic modifications and the 3D genome, as well as the role of 3D genome architecture in gene regulation. As previously mentioned, several single‐cell assays have been developed to simultaneously probe 3D genomics and other omics data.^[^ ^34^, ^57^, ^58^ ^]^ However, the development of computational tools to analyze these data remains limited. By training a neural network to predict gene expression in single cells using scCAFE embeddings, it might be possible to apply interpretable deep learning techniques to identify the most critical sub‐structures in the 3D genome organization responsible for specific gene expression states. This conceptual framework would be constructed on the strong foundation established by our scCAFE embeddings, which have demonstrated exceptional presentational capabilities.

Moreover, genome architecture varies widely across species and cell cycle stages, as highlighted by studies on Rabl‐like conformations in different evolution stages^[^ ^60^, ^61^ ^]^ and chromatin transition trajectory during mitosis.^[^ ^62^ ^]^ While scCAFE is optimized for single‐cell Hi‐C data, its application to these contexts could yield valuable insights once suitable single‐cell datasets become available. Expanding analyses to diverse genome architectures and cell cycle stages represents an exciting direction for future research.

## 4 Experimental Section

### Data Representation and Model Architecture of scCAFE

The data representation method in scCAFE was inherited from our previous work scGSLoop.^[^ ^28^ ^]^ In particular, each cis‐contact map of a chromosome within a cell was represented as a graph, where each genomic bin served as a node, and the contacts between bins were represented by edges connecting these nodes. Leveraging the multi‐view nature of Hi‐C data,^[^ ^28^, ^31^ ^]^ the node features were extracted from DNA sequence information by encoding k‐mer and motif occurrences as vectors. Given a scHi‐C dataset with *L* cells and *C* chromosomes per cell, the data can be mathematically represented as:

(1) $$\begin{array}{cc} G^{\left(l,c\right)} & =\left[V^{\left(l,c\right)},E^{\left(l,c\right)}\right] \end{array}$$

(2) $$\begin{array}{cc} X^{\left(l,c\right)} & \in R^{N\times F} \end{array}$$

where *l* denotes the index of the cell in the dataset, and *c* denotes the *c*‐th chromosome in the cell. *V* ^(*l*, *c*)^ and *E* ^(*l*, *c*)^ represent the node set (genomic bins) and edge set (contacts) of the graph, respectively. Each row $x_{i}^{\left(l,c\right)}$ in *X* ^(*l*, *c*)^ represents the feature vector of node *i* in the chromosome. All graphs were constructed using 10 kb‐resolution contact maps.

In scCAFE, we employed a variational graph autoencoder (VGAE)^[^ ^63^ ^]^‐like neural network to capture the high‐level features hidden within the contact maps. We utilized a multi‐objective loss function to optimize both the reconstruction of the original contact maps and the prediction of chromatin loops. Compared to scGSLoop,^[^ ^28^ ^]^ the auxiliary task of contact map reconstruction in scCAFE enabled the model to learn a more comprehensive representation of the chromatin organization, thereby allowing the prediction of multi‐scale architectural features. Specifically, we treated the node embeddings in each graph as a sequence and applied unsupervised algorithms to detect higher‐order organizational patterns in the genome, including TLDs and compartments.

Similar to scGSLoop, the scHi‐C contact maps with low quality were enhanced using a k‐nearest neighbors (KNN) algorithm before being input to the VGAE module. Initially, the cells were projected into a lower‐dimensional space using scHiCTools.^[^ ^48^ ^]^ The contact matrices of each cell and its three nearest neighbors in this lower‐dimensional space were averaged to create the input graphs for the VGAE.

### Multi‐Task VGAE for Loop Calling and Node Representation Learning

In our approach, we employed a multi‐task VGAE for node representation learning. We simultaneously optimized the VGAE for two related tasks: loop prediction and contact reconstruction. Both tasks involved edge‐level predictions. For the loop prediction task, the model was trained to distinguish between positive looping node pairs, as specified in the reference loop list, and an equal number of negative non‐looping pairs, which were sampled to match the count of the positive pairs. For the reconstruction task, the positive examples were the edges existing in the contact maps, and the negative samples were generated by randomly selecting non‐existent edges within the contact maps. In both tasks, the proximity‐aware negative sampling mechanism^[^ ^28^ ^]^ was utilized to improve the model's decision boundary and, consequently, enhance its learning capability.

The VGAE we adopted in this study was composed of a GraphSage^[^ ^64^ ^]^ encoder and two edge‐level dense decoders with identical architecture, each dedicated to a specific task. The forwarding rules can be formulated as follows:

(3) $$\begin{array}{cc} \mu_{u},\log\sigma_{u} & =E\left(G,X\right) \end{array}$$

(4) $$\begin{array}{cc} z_{u} & =\mu_{u}+\sigma_{u}⊙\varepsilon \end{array}$$

(5) $$\begin{array}{cc} {\hat{y}}_{uv}^{\mathrm{recon}} & =\Phi \left(\left[z_{u},z_{v}\right]\right) \end{array}$$

(6) $$\begin{array}{cc} {\hat{y}}_{uv}^{\mathrm{loop}} & =\Theta \left(\left[z_{u},z_{v}\right]\right) \end{array}$$

where E, Φ, and Θ are the encoder, reconstruction decoder, and loop decoder, respectively. ϵ denotes a Gaussian distribution such that $\varepsilon ∼N\left(0,I\right)$. *u* and *v* are indices of nodes in a chromosome graph, and [·, ·] represents the concatenation operation. In both tasks, the loss functions used were binary cross‐entropy. The total loss was the sum of these binary cross‐entropy losses and the KL loss.

The model trained in this multi‐task manner can directly predict loops during the inference stage. Additionally, it can generate latent embeddings that can be utilized downstream for TLD and compartment detection.

### Detection of TLDs and Compartments

The detection of single‐cell TLDs and compartments was achieved using unsupervised learning methods. Here, the node embeddings within a chromosome graph, generated by the encoder E, were treated as a linearly connected sequence corresponding to the genomic bins' positions in the linear genome. For TLD pattern identification, we first reduced the dimensionality of embeddings using principal component analysis (PCA). We then applied hierarchical clustering with a connectivity constraint to determine the divisions of TLDs. This connectivity constraint ensured that the nodes within each resulting cluster were connected consecutively, preserving the sequential characteristics of the genomic bins in TLDs. The connectivity matrix $M^{\left(l,c\right)}\in R^{N\times N}$ for each chromosome graph $G^{\left(l,c\right)}$ is defined as:

(7) $$M_{uv}^{\left(l,c\right)}=\left\{\begin{array}{cc} 1 & \mathrm{if}v=u+1\mathrm{or}u=v+1\\ 0 & \mathrm{otherwise} \end{array}\right.$$

The number of clusters for each chromosome was calculated by dividing the chromosome length by 200 kb. In this way, a set of candidate TLD boundaries was identified. These candidate boundary bins were further categorized into two groups using KMeans clustering. The group with a higher number of CTCF motifs was selected as the final list of TLD annotations.

Single‐cell compartments were identified using a hidden Markov model (HMM). By modeling the genomic bins in each chromosome as a sequence, we assumed that two hidden states underlie the observations, corresponding to compartments A and B. The HMM model was trained on the node embeddings of 100 chromosomes. The trained model was then used to predict the hidden states of nodes for each graph in the dataset. We subsequently adjusted the signs of the posterior probabilities based on GC content. Specifically, the state with higher GC content was assigned positive probability values, while the state with lower GC content was assigned negative probability values. This sign flipping scheme was consistent with the practices in previous studies^[^ ^6^, ^65^, ^66^ ^]^ and was also adopted in existing Hi‐C analysis libraries.^[^ ^67^ ^]^

Both TLDs and compartments were detected at the resolution of 10 kb. The resolution of the predictions was then coarsened to meet the requirements for downstream analysis as needed.

### Time Complexity of Connectivity‐Constrained Hierarchical Clustering in scCAFE

Hierarchical clustering is a common approach for identifying biologically meaningful structures in single‐cell data. However, traditional agglomerative hierarchical clustering has a high computational complexity of *O*(*n* ^3^), where *n* is the number of data points. This complexity arises from evaluating all pairwise distances and updating the distance matrix during each merge step. To address this, scCAFE employs a *connectivity‐constrained hierarchical clustering* model, which significantly reduces computational cost by restricting merges to adjacent genomic regions.

The time complexity of the standard agglomerative clustering can be broken down as follows:

1. Initialization: Compute pairwise distances for all *n* data points, requiring *O*(*n* ^2^) operations.
2. Iterative Merging: Perform *n* − 1 merge steps, where each step involves:
  - Finding the minimum distance in the distance matrix: *O*(*n* ^2^).
  - Updating the distance matrix for the new cluster: *O*(*n*).

Since each of the *n* − 1 merge steps requires *O*(*n* ^2^) operations, the total complexity is *O*(*n* ^3^).

In scCAFE, a connectivity constraint was imposed so that the computational complexity was significantly reduced. Specifically, we restrict merging to adjacent regions along the linear genome, as TLDs are biologically required to consist of contiguous genomic regions. This is achieved by constructing a connectivity matrix *A*, defined as:

(8) $$A=\left[\begin{array}{cccccc} 0 & 1 & 0 & 0 & \cdots & 0\\ 1 & 0 & 1 & 0 & \cdots & 0\\ 0 & 1 & 0 & 1 & \cdots & 0\\ 0 & 0 & 1 & 0 & \cdots & 0\\ \vdots & \vdots & \vdots & \vdots & \ddots & 1\\ 0 & 0 & 0 & 0 & 1 & 0 \end{array}\right]$$

This matrix limits candidate pairs for merging to *n* − 1, corresponding to adjacent bins. As a result:

- Finding the minimum distance is reduced to *O*(*n*), as it only requires evaluating *n* − 1 pairs.
- The iterative merging process, which involves *n* − 1 steps, now has an overall complexity of *O*(*n* ^2^).

Thus, the total time complexity of the connectivity‐constrained hierarchical clustering model is reduced to *O*(*n* ^2^), making it significantly more efficient than standard hierarchical clustering.

### Dynamic Parameter Selection Using TLD Size Estimation

We developed a dynamic parameter selection mechanism for determining the optimal average TLD size. This approach enhances the generalizability of the model by aligning the generated TLD size distribution with biologically relevant reference bulk data.

The dynamic selection algorithm leverages a reference TAD annotation track derived from bulk Hi‐C data and iteratively searches for the average TLD size that minimizes the dissimilarity between the generated and reference TAD size distributions. The algorithm begins by randomly sampling a subset of chromosome graphs from the dataset. It then evaluates candidate average TLD sizes, ranging from 5 to 100 (bins). For each candidate size, the algorithm computes a dissimilarity score using the mean Wasserstein distance (Earth Mover's Distance) between the reference and generated size distributions:

(9) $$D=\frac{\sum_{i=1}^{C}W\left(\mu_{i},\nu_{i}\right)}{C}$$

Here, *W* denotes the first‐order Wasserstein distance, *C* is the number of chromosome graphs in the subsampled dataset, and µ_*i*_ and ν_*i*_ represent the generated and reference TAD size distributions, respectively. The optimal average TLD size, 〈TLD〉*, is selected by minimizing the dissimilarity score:

(10) $${\left\langle \mathrm{TLD}\right\rangle }^{*}={\mathrm{argmin}}_{\left\langle \mathrm{TLD}\right\rangle }D$$

This adaptive approach ensures that the resulting TLD size distribution better reflects the underlying biological variability and is less sensitive to manual parameter selection.

Users can choose between using a fixed average TLD size or enabling the adaptive selection algorithm. In this study, for the mESC dataset, the optimal 〈TLD〉* was determined to be approximately 20 bins (Figure S7a, Supporting Information). Similarly, for the ΔRegions dataset, the optimal value was also near 20 bins (Figure S7b, Supporting Information). To ensure consistency and allow for a straightforward comparison of the effects of deleted genomic regions, a fixed value of 〈TLD〉 = 20 was used for experiments on the complete and ΔRegions datasets.

### Prediction and Evaluation of Chromatin Loops in Ancient Mammoth 3D Genome

To further evaluate the versatility of scCAFE, we applied it to analyze Hi‐C data derived from mammoth ancient DNA.^[^ ^39^ ^]^ We compared its performance against several widely used Hi‐C analysis tools, including SIP,^[^ ^35^ ^]^ cooltools,^[^ ^36^ ^]^ Peakachu,^[^ ^37^ ^]^ and Chromosight,^[^ ^38^ ^]^ focusing on its ability to predict chromatin loops in ancient DNA Hi‐C datasets. A comparative analysis was conducted by generating ROC curves to assess the classification performance of each method on this challenging dataset. The predictions were made using the scCAFE model trained on the mES dataset.

The reference loops used to evaluate the model's performance on the ancient mammoth dataset were derived from a modern African elephant (*Loxodonta africana*, LA) Hi‐C dataset.^[^ ^39^ ^]^ These loops were mapped to the same genome assembly as the mammoth dataset (MamPri_Loxafr3.0_assisted_HiC). To identify chromatin loops in the LA contact maps, we employed FitHiC^[^ ^68^, ^69^ ^]^ and SIP,^[^ ^35^ ^]^ subsequently merging the results to create the final reference loop list.

Since the mammoth dataset contains only a single, extremely sparse contact map for each chromosome, the predictions were insufficient to generate a complete PR curve that provides meaningful insights. Instead, we employed an ROC curve to illustrate the model's predictive power based on the existing entries in the contact maps. In this way, the metric highlights the differences in the capabilities of various callers in discriminating loops from other contacts.

### Consensus Architectural Feature Prediction

scCAFE also provides methods for the consensus architectural features of a cell population, such as cell type or subtype. For consensus loop detection, we followed the simple aggregation method used in ref. [28], which calculates the average loop probability across the cell population.

To obtain the consensus TLD predictions, a more complex method is required. First, a co‐association matrix was created by recording the number of cells in which each pair of nodes was assigned to the same cluster. This co‐association matrix was then divided by the total number of cells, resulting in an adjacency matrix. The adjacency matrix was utilized to obtain the spectral embedding. Finally, hierarchical clustering was applied to the spectral embedding, effectively dividing the nodes within a chromosome into multiple TLD regions.

For the consensus predictions of compartments, we utilized the GC‐flipped posterior probabilities of the loci and calculated the average across all cells within the population.

### Embedding and Classification Using Latent and Architectural Features

We examined the predictive power of different sets of features (including the latent high‐level features and single‐cell architectural features) in determining cell identity in the human prefrontal cortext (hPFC) dataset. This was achieved by visualizing the 2D embeddings of cells and conducting supervised classification on the cells.

To create the latent high‐level features that can fit into memory, we first employed PCA to reduce the dimensionality of the output from the encoder E of scCAFE. Only the first principal component was retained in our study, resulting in each chromosome graph having a single feature vector. This chromosome feature vector was then pooled by summing up every 10 dimensions, so that the final dimensionality was consistent with the number of bins in the 100 kb‐resolution contact map.

Architectural features were also aligned to 100 kb resolution. Loop features were generated by taking the numbers of loops associated with each 100 kb anchor. The presence of TLD boundaries in 100 kb bins was converted into a binary vector, which was subsequently used as the TLD features. Compartment features were the posterior probabilities from HMM coarsened to 100 kb resolution by taking the average of every 10 entries. The latent high‐level features, loop features, TLD features, and compartment features were all at 100 kb resolution, ensuring they have the same dimensionality.

Each set of features was embedded into a 2D space using a combination of PCA and UMAP. PCA initially reduced the dimensionality, and then UMAP projected the lower‐dimensional representations into 2D for visualization. Supervised machine learning method was then used to quantify the discriminative power of the features. In particular, seven linear SVMs with L1 regularization were trained for each set of features to classify the cells into seven types. The training set for each feature set was composed of 75% of the cells, and the left cells were used for testing the model's performance.

### Discovery of Marker Loop Anchors

Combining traditional machine learning techniques and statistical methods, we identified marker loop anchors for the seven cell types in the hPFC dataset. Different from the encoding scheme described in the previous section, we took the loop anchors of 10 kb resolution, and subsequently encoded them utilizing the tf‐idf scheme. A total of seven linear SVM models were trained using these features. Each model was specifically optimized to classify cells of a particular cell type from the remaining cells in the dataset. The learned weights of each SVM were then considered as the contributions of the features (anchor loci) to the classification outcomes. We selected the top 50 contributing features with the highest positive weights for each cell type. The de‐duplicated union of these loci, identified across all cell types, constitutes the pool of candidate markers. Scanpy^[^ ^54^ ^]^ was utilized to further identify marker loop anchors for each cell type by performing statistical tests across cell types. Among the loci in the candidate marker pool, it identified those that are associated with a significantly higher number of chromatin loops compared to other cell types. This analysis was based on the assumption that the distribution of the number of associated loops is similar to that of the expression volume of RNA, which was modeled in Scanpy. We selected the default method for statistical test (t‐test) in Scanpy, and the correction method was Benjamini–Hochberg.

The classification performance using these tf‐idf features was also evaluated and is reported in this manuscript.

### Reference Loop Lists

For the evaluation of our method, we used reference loop lists derived from previously published studies:

hPFC and mESC datasets: The reference loops for these datasets were obtained directly from the SnapHiC study.^[^ ^22^ ^]^ Specifically: For mESC, the reference loops were the union of MAPS^[^ ^70^ ^]^‐identified loops from H3K4me3 PLAC‐seq, cohesin HiChIP, and H3K27ac HiChIP datasets, called at 10 kb resolution. For hPFC, the reference loops were MAPS‐identified loops from bulk H3K4me3 PLAC‐seq data of oligodendrocytes, microglia, and neurons.

scNanoHiC datasets: We followed the protocol from [16] to generate the reference loops for the scNanoHiC datasets. The first part of the reference loops were derived from bulk in situ Hi‐C data, processed using HiCCUPS at 10 kb resolution with default parameters. Additional reference loops from H3K27ac and cohesin HiChIP in GM12878 cells, as well as H3K27ac, cohesin HiChIP, and SMC1 ChIA‐PET in mESC, were taken from the FitHiChIP study.^[^ ^71^ ^]^

Ancient Mammoth 3D Genome: The reference loops for the ancient mammoth dataset were obtained from a modern African elephant (*Loxodonta africana*) Hi‐C dataset. Loops were identified using both FitHiC^[^ ^68^, ^69^ ^]^ and SIP,^[^ ^38^ ^]^ and the results were merged to form the final reference loop list. These loops were mapped to the same genome assembly as the mammoth dataset (MamPri_Loxafr3.0_assisted_HiC).

### Metrics for Performance Evaluation

In this study, we employed F1 score and precision‐recall curve (PR‐curve) to evaluate the models' performance in terms of loop calling. The F1 score is a metric that effectively combines precision and recall into a single value, striking a balance between these two measures. However, calculating the F1 score requires choosing a cutoff point to convert the probabilities generated by neural networks into binary predictions. Consequently, this metric may be less suitable when an extensive evaluation across all thresholds is required. To overcome this issue, we additionally assessed the performance of the models using PR‐curve for a comprehensive evaluation.

The metrics were calculated based on the entries within the genomic distances ranging from 100 kb to 1 Mb on the contact maps, which corresponded to the regions where the predictions were made. It is worth noting that, different from the slack F1 score used in refs. [28] and [22], here we adopted the original definition of precision and recall to calculate F1 score and plot the PR‐curve.

The metrics for evaluating the cell type classification results include the F1 score, accuracy, and the confusion matrix. Given the multiclass classification setting, we calculated the macro‐F1 scores to assess performance. Accuracy measures the overall correctness of the classification, while the confusion matrix provides a visual representation of the algorithm's detailed performance.

### Architectural Features Called From Bulk Hi‐C Data

We used the same reference loop lists for the mESC dataset and the hPFC dataset as the ones used in SnapHiC^[^ ^22^ ^]^ and scGSLoop.^[^ ^28^ ^]^ The reference loops were called from bulk Hi‐C data and were used as labels for training the model and evaluating the model's loop predictions.

The insulation scores of genomic bins were calculated from bulk Hi‐C data using the insulation function in FAN‐C.^[^ ^67^ ^]^ These scores were obtained with window sizes of 1, 1.5, 2, 2.5, 3, 3.5, and 4 Mb. Per‐chromosomal normalization and log transformation were applied to the scores.

To generate the bulk A/B compartment annotations, we applied eigendecomposition on the Pearson correlation matrix of each contact map at 100 kb resolution. The resulting first eigenvalues were then flipped using GC content such that the entries in A compartment have positive values, while entries in the B compartment have negative values. This A/B compartment calling process was performed using the compartments function in FAN‐C package.

### Aggregate Analysis

The aggregate analyses of loops, TLDs, and compartments were also carried out using FAN‐C.^[^ ^67^ ^]^ For the loop aggregate analysis, we selected the top 5000 loops with the highest predicted probabilities, and aggregated the looping pixel and the surrounding pixels. The TLD aggregation was conducted following the method in ref. [14]. The contacts within TLDs and their surrounding regions were cropped into square matrices and re‐scaled to a uniform size. These matrices were subsequently piled up to produce the final aggregate figure. We employed a saddle plot to create the aggregate profile for compartments. Each entry on the plot was the average O/E contact between the regions represented by the row and those represented by the corresponding column.

All aggregate analyses were conducted on the same bulk Hi‐C data, utilizing annotations derived from the single‐cell dataset (including both single‐cell annotations and consensus annotations). This approach guaranteed the consistency of data sources for aggregation and ensured the comparability of the aggregate results. For TLD aggregation, only TLDs of size larger than 100 kb and smaller than 1 Mb were considered.

### Training Setups and Strategies for Mitigating Overfitting

To ensure that our model achieves generalizability while avoiding overfitting, we employed several strategies and carefully tuned our training hyperparameters. A key factor in the design of our evaluation framework was the use of a cross‐species test dataset, which allowed us to rigorously assess the model's ability to generalize across different species. Additionally, we implemented the following regularization techniques and training strategies:

1. Regularization Techniques: To prevent overfitting during training, we applied dropout and weight decay:
  - Dropout: A dropout rate of 0.2 was used in the decoder layers to reduce reliance on specific neurons and improve generalization.
  - Weight Decay: L2 regularization with a weight decay factor of 1 × 10^−3^ was applied to penalize large weights and constrain model complexity.
2. Validation Strategy and Early Stopping: We utilized independent chromosomes from the same species as a validation set. This ensured that the model was evaluated on unseen data during training, promoting generalization beyond the training data. Early stopping was employed to halt training when the validation loss did not improve for five consecutive epochs, with a threshold of less than 1 × 10^−6^ for loss decrease.
3. Training Hyperparameters: Further details of the model architecture and training hyperparameters are as follows:
  - Model Architecture:
    – Encoder: The encoder was designed using three GraphSAGE layers:
      * The first layer encoded node features into 128‐dimensional vectors with ReLU activation.
      * The second layer computed the mean of the latent distribution *q* with 64 channels and mean aggregation.
      * The third layer computed the log‐standard deviation of *q* with 64 channels and mean aggregation.
    – Decoders: Both the reconstruction decoder and the loop classification decoder shared the same architecture, consisting of 4 linear layers with output channels [96, 64, 16, 1] and ReLU activation. A dropout rate of 0.2 was applied after each layer.
  - Loss Function: The loss function combined binary cross‐entropy loss with KL divergence to balance reconstruction accuracy and latent space regularization.
  - Learning Rate: A learning rate of 2 × 10^−5^ was used, optimized for stable convergence.

By combining these strategies, we ensured that the model was robust to overfitting and capable of generalizing across species, as demonstrated by its performance on the cross‐species evaluation dataset.

### Statistical Analysis

In this study, we employed SciPy to conduct statistical tests. Wilcoxon signed‐rank test was used to compare the F1 scores, Precisions, and Recalls of single‐cell loops predicted by different methods. Other comparisons for independent groups in this manuscript were conducted using Mann–Whitney U rank test.

In all statistical analyses, * represents a p‐value less than 0.05, indicating statistical significance. Specifically, ** denotes p‐values less than 0.01, and *** denotes p‐values less than 0.001. All tests were two‐sided.

### Data and Code Availability

The code of scGSLoop is open‐source and publicly available on GitHub at https://github.com/fzbio/scCAFE.

The data used in this study are all publicly available. No new data were generated in this study. The mESC scHi‐C data, initially generated by Nagano et al.,^[^ ^12^ ^]^ are accessible via the Gene Expression Omnibus (GEO) with the accession number GSE94489. Raw data for the hPFC dataset^[^ ^34^ ^]^ are available on GEO under accession number GSE130711. For the two datasets, we used the processed contact maps which are available at https://github.com/tanaylab/schic2 and https://salkinstitute.app.box.com/s/fp63a4j36m5k255dhje3zcj5kfuzkyj1/folder/82403061106, respectively. The scNanoHiC datasets were downloaded from GEO with accession number GSE217189. The ancient woolly mammoth dataset and the modern African elephant Hi‐C, as well as the genome assembly, were obtained from GEO GSE268050. CTCF motif annotations for mm10 and hg19 were collected and processed from.^[^ ^72^ ^]^ These annotations were generated by scanning the whole genomes for CTCF motifs with FIMO.^[^ ^73^ ^]^

The genomic coordinates and annotations for the detected loops, TLDs, and compartments generated in this study have been made publicly available for download at Zenodo under the accession number https://doi.org/10.5281/zenodo.15006290.

## Conflict of Interest

The authors declare no conflict of interest.

## Supporting information

Supporting Information

## Data Availability

The data that support the findings of this study are available from the corresponding author upon reasonable request.
