# Supplementary material for: Unveiling Multi‐Scale Architectural Features in Single‐Cell Hi‐C Data Using scCAFE
Source: Adv Sci (Weinh). 2025 Apr 24;12(23):2416432. doi: 10.1002/advs.202416432 (PMC12199332; doi:10.1002/advs.202416432)
Supplement: Supplementary file 1 — Supporting Information [file ADVS-12-2416432-s001.pdf]

ADVANCED SCIENCE
Open Access

Supporting Information

for *Adv. Sci.*, DOI 10.1002/advs.202416432

Unveiling Multi-Scale Architectural Features in Single-Cell Hi-C Data Using scCAFE

Fuzhou Wang, Jiecong Lin, Hamid Alinejad-Rokny, Wenjing Ma, Lingkuan Meng, Lei Huang, Jixiang Yu, Nanjun Chen, Yuchen Wang, Zhongyu Yao, Weidun Xie, Ka-Chun Wong and Xiangtao Li**

Supplementary Figures

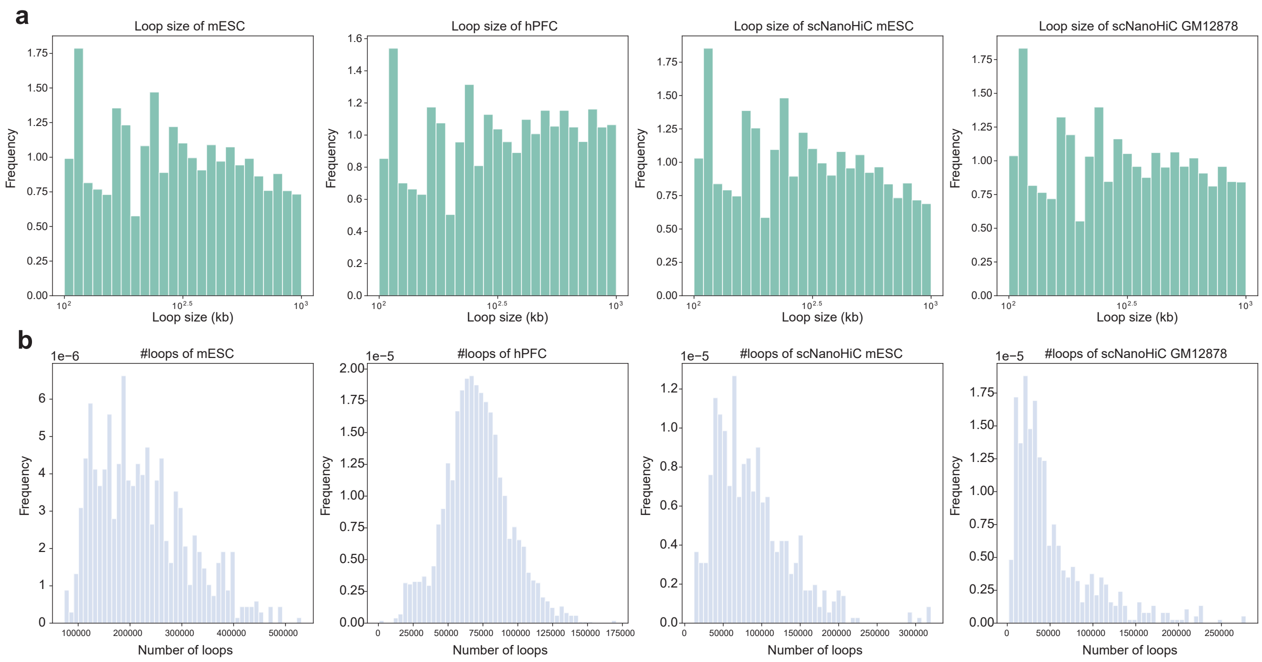

Figure S1. Statistical properties of single-cell loops predicted by scCAFE in the datasets of mESC, hPFC, scNanoHiC mESC, and scNanoHiC GM12878. **(a)** Distribution of loop sizes. **(b)** Distribution of the number of loops in different cells.

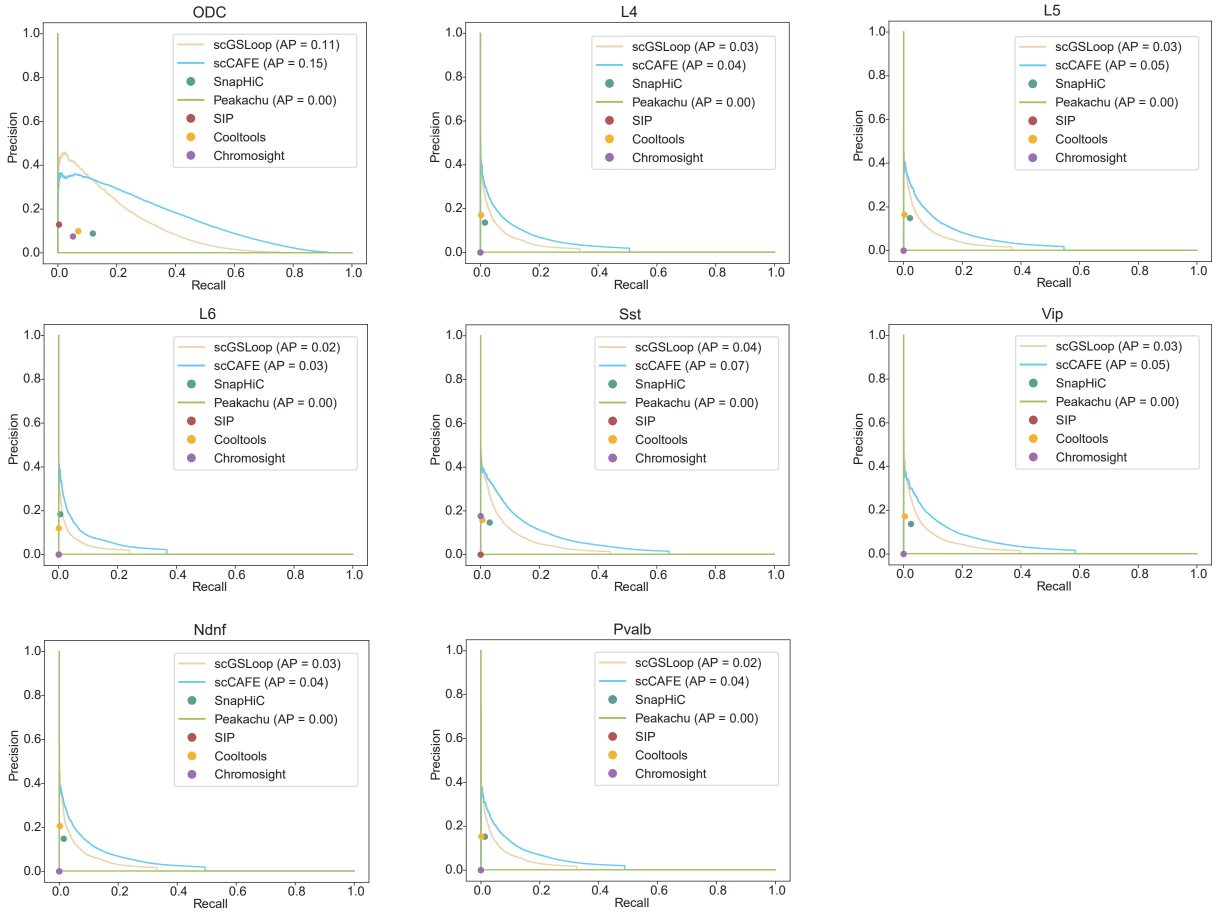

Figure S2. Precision-recall plots of the consensus loops in ODC, L4, L5, L6, Sst, Vip, Ndnf, Pvalb cells predicted by scGSLoop, scCAFE, SnapHiC, Peakachu, SIP, Cooltools, and Chromosight.

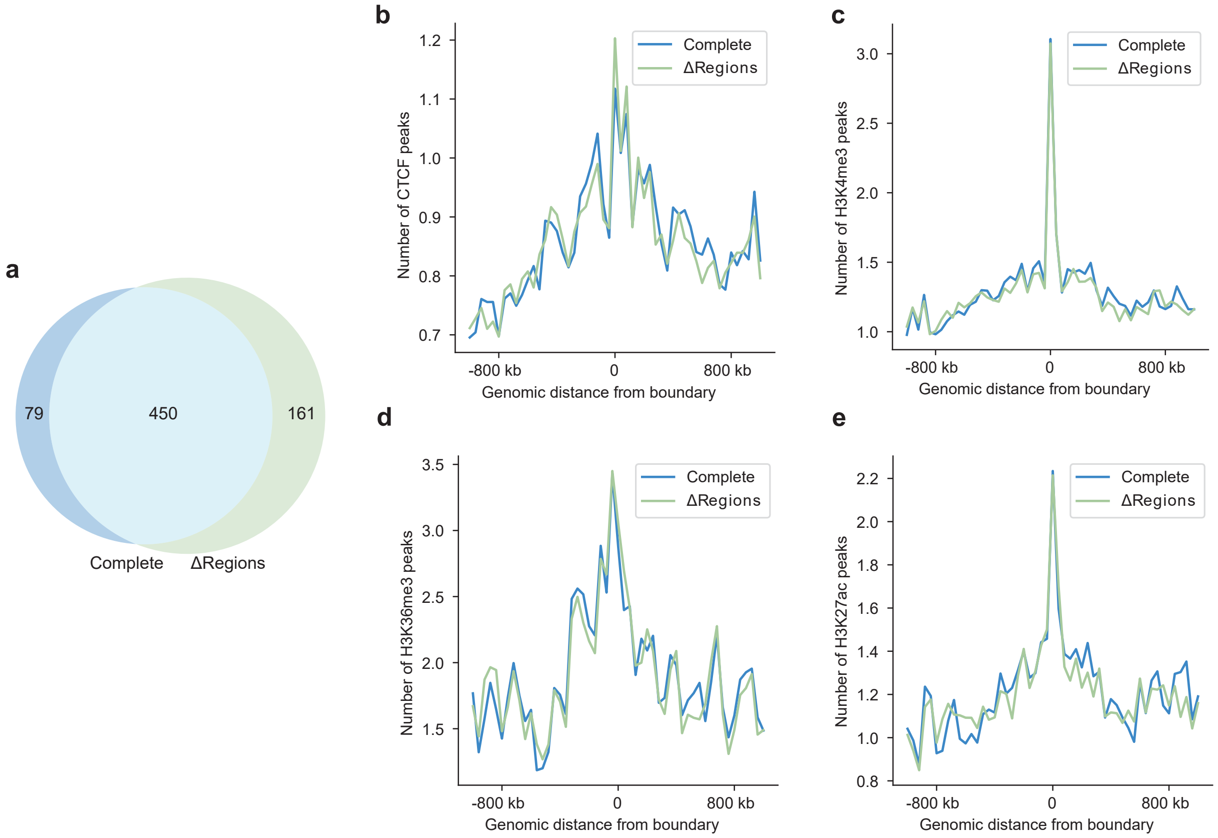

Figure S3. Comparison of TLD predictions between the complete dataset and the △Regions dataset with artificially deleted regions. **(a)** Venn diagram illustrating the overlap of predictions between the complete dataset and the △Regions dataset. **(b)** CTCF enrichment profiles for the complete and △Regions datasets. **(c)** H3K4me3 enrichment profiles for the two datasets. **(d)** H3K36me3 enrichment profiles for the two datasets. **(e)** H3K27ac enrichment profiles for the two datasets.

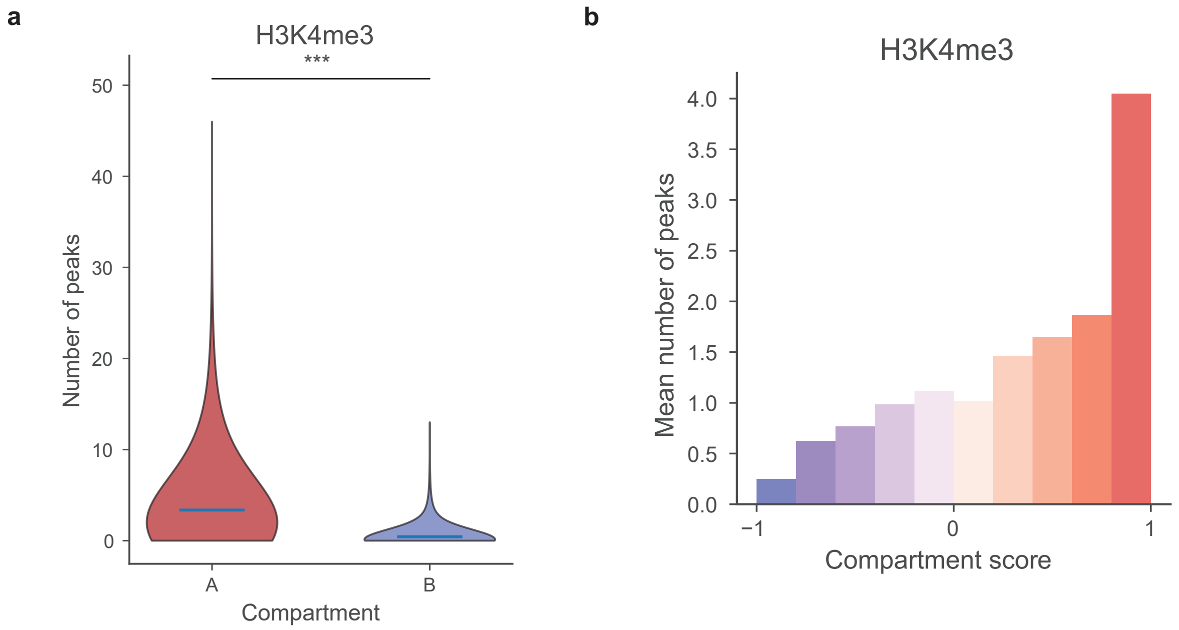

Figure S4. (a) Numbers of H3K4me3 ChIP-Seq peaks in compartment A and B. **(b)** Numbers of H3K4me3 peaks at different scCAFE compartment scores.

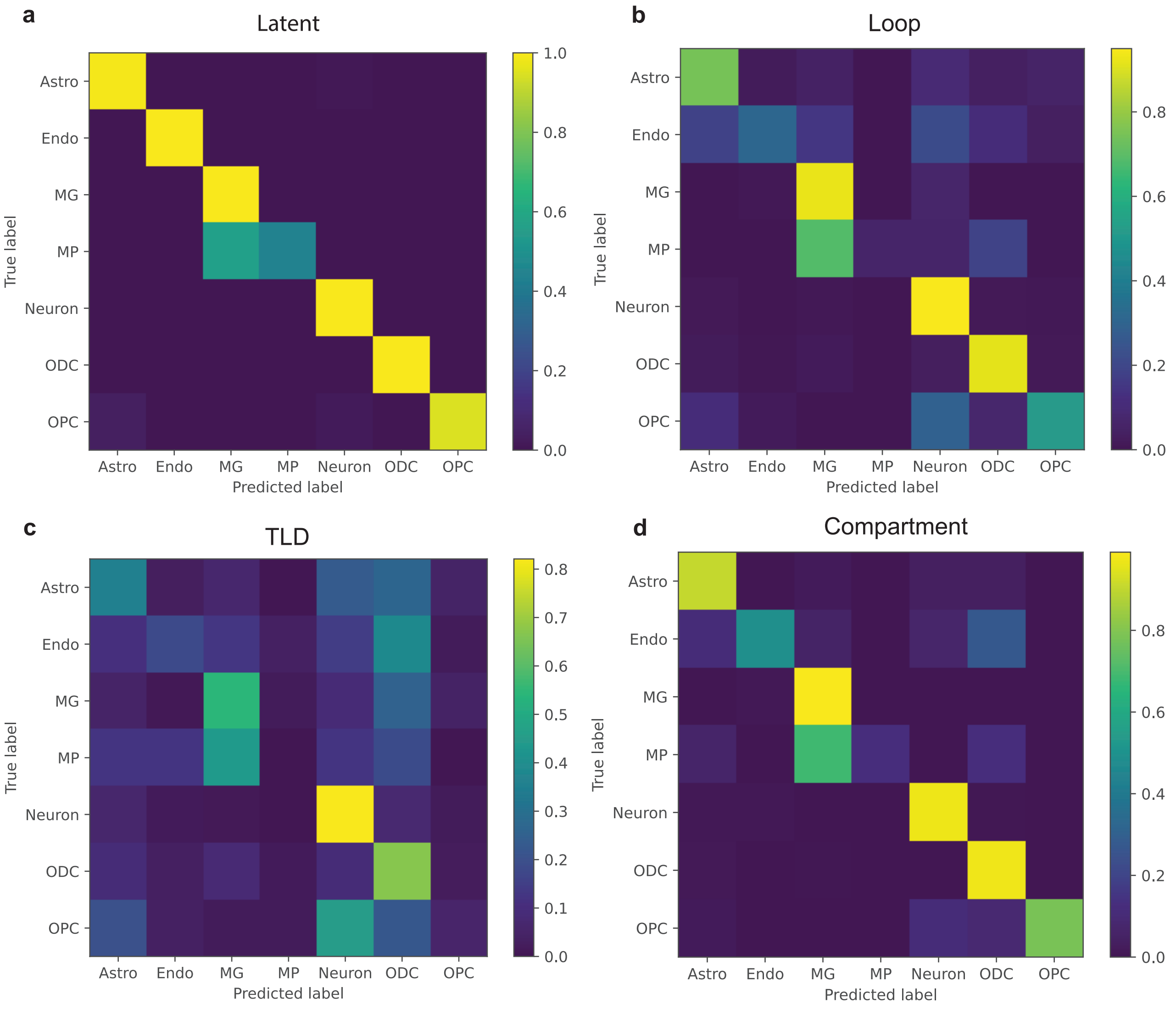

Figure S5. Confusion matrices of different sets of architectural features. **(a)** scCAFE latent features. **(b)** Single-cell loop features. **(c)** Single-cell TLD features. **(d)** Single-cell compartment features.

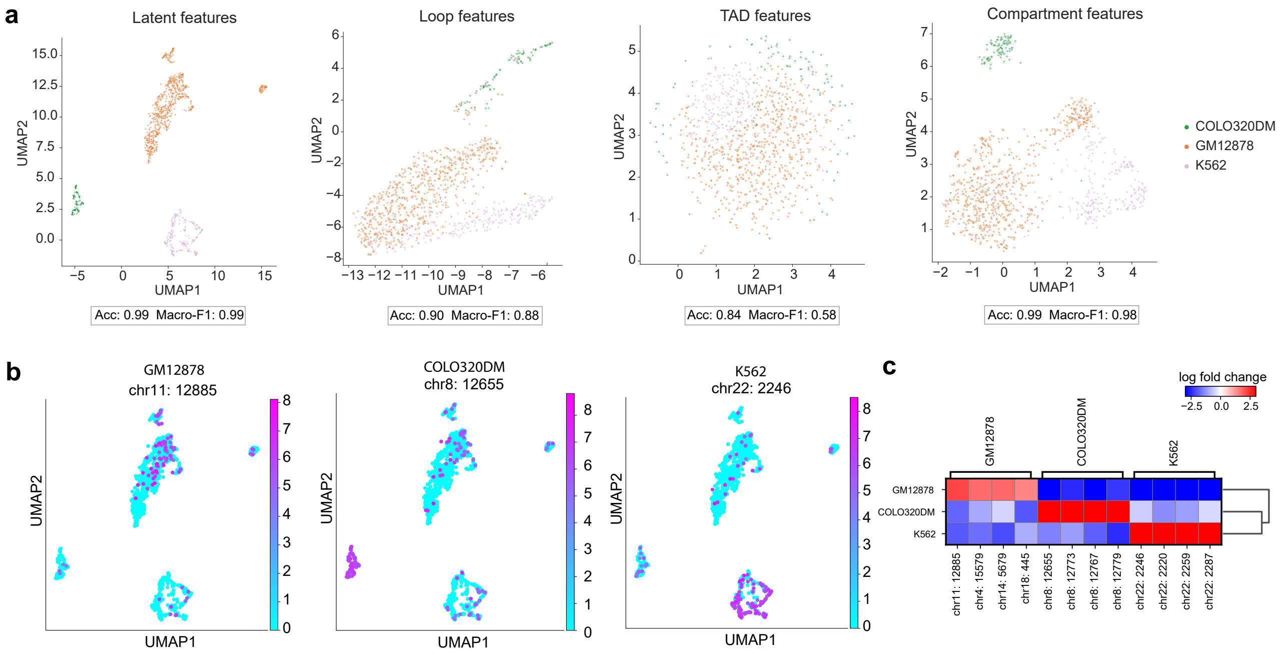

Figure S6. Predictive ability of different levels of architectural features on classifying cell lines from the scNanoHiC dataset. **(a)** UMAP plots of scCAFE latent features, single-cell loops, TLDs, and compartments on the scNanoHiC dataset. **(b)** "Marker loop anchors" identified by scCAFE in GM12878, COLO320DM, and K562. **(c)** Matrix plot of marker loop anchors. Each row represents a cell line, and each column corresponds to a loop anchor region in the genome. The color of each entry denotes the log fold change in the number of loops compared to other cell lines.

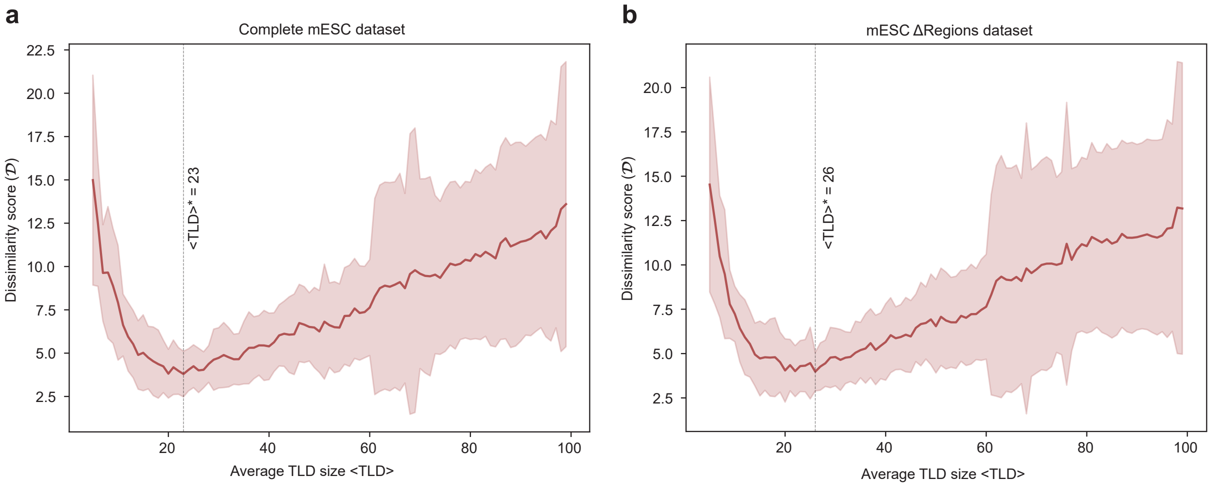

Figure S7. Dissimilarity scores at various average TLD size $\langle \mathrm{TLD} \rangle$ in the complete dataset and the ΔRegions dataset. **(a)** Dissimilarity score trend in the complete dataset. **(b)** Dissimilarity score trend in the ΔRegions dataset.
